# A Curcumin Derivative, 2,6-Bis(2,5-dimethoxybenzylidene)-cyclohexanone (BDMC33) Attenuates Prostaglandin E_2_ Synthesis via Selective Suppression of Cyclooxygenase-2 in IFN-γ/LPS-Stimulated Macrophages

**DOI:** 10.3390/molecules16119728

**Published:** 2011-11-23

**Authors:** Ka-Heng Lee, Faridah Abas, Noorjahan Banu Mohamed Alitheen, Khozirah Shaari, Nordin Haji Lajis, Syahida Ahmad

**Affiliations:** 1 Department of Biochemistry, Faculty of Biotechnology and Biomolecular Sciences, Universiti Putra Malaysia, 43400 UPM Serdang, Selangor, Malaysia; Email: heng1011@yahoo.co.uk (K.-H.L.); 2 Institute of Biosciences, Universiti Putra Malaysia, 43400 UPM Serdang, Selangor, Malaysia; 3 Department of Food Science, Faculty of Food Science and Technology, Universiti Putra Malaysia, 43400 UPM Serdang, Selangor, Malaysia; Email: faridah@food.upm.edu.my (F.A.); 4 Department of Cell and Molecular Biology, Faculty of Biotechnology and Biomolecular Sciences, Universiti Putra Malaysia, 43400 UPM Serdang, Selangor, Malaysia; Email: noorjahan@biotech.upm.edu.my (N.B.M.A.); mdnordin@science.upm.edu.my (N.H.L.); 5 Department of Chemistry, Faculty of Sciences, Universiti Putra Malaysia, 43400 UPM Serdang, Selangor, Malaysia; Email: khozirah@science.upm.edu.my (K.S.)

**Keywords:** anti-inflammatory, BDMC33, cyclooxygenase, PGE_2_, RAW264.7

## Abstract

Our preliminary screening had shown that the curcumin derivative [2,6-bis(2,5-dimethoxybenzylidene)cyclohexanone] or BDMC33 exhibited improved anti-inflammatory activity by inhibiting nitric oxide synthesis in activated macrophage cells. In this study, we further investigated the anti-inflammatory properties of BDMC33 on PGE_2 _synthesis and cyclooxygenase (COX) expression in IFN-γ/LPS-stimulated macrophages. We found that BDMC33 significantly inhibited PGE_2_ synthesis in a concentration-dependent manner albeit at a low inhibition level with an IC_50_ value of 47.33 ± 1.00 µM. Interestingly, the PGE_2_ inhibitory activity of BDMC33 is not attributed to inhibition of the COX enzyme activities, but rather BDMC33 selectively down-regulated the expression of COX-2. In addition, BDMC33 modulates the COX expression by sustaining the constitutively COX-1 expression in IFN-γ/LPS-treated macrophage cells. Collectively, the experimental data suggest an immunodulatory action of BDMC33 on PGE_2_ synthesis and COX expression, making it a possible treatment for inflammatory disorders with minimal gastrointestinal-related side effects.

## Abbreviations

BDMC33: 2,6-bis(2,5-dimethoxybenzylidene)cyclohexanone
COX: cyclooxygenase
DMSO: dimethyl sulfoxide
DXM: dexamethasone
FBS: foetal bovine serum
HRP: horseradish peroxidase
IFN-γ: interferon-gamma
LPS: lipopolysaccharide
NO: nitric oxide
PGE_2_: prostaglandin E_2_
NSAID: non-steroidal anti-inflammatory drug
RAW 264.7: murine macrophage cells

## 1. Introduction

Prostaglandins (PG) are potent bioactive lipid molecules derived from arachidonic acid (AA) and produced by nearly all cells within the body. The most abundant PG in the human body is PGE_2_, which regulates several responses in the humans such as the reproductive, gastrointestinal, neuroendocrine, and immune systems [1]. The biosynthesis of PGE_2_ is catalyzed by a rate-limiting enzyme namely cyclooxygenase (COX), also known as prostaglandin endoperoxide H_2_ (PGH_2_) synthase [2]. To date, three isoform of COX have been identified. Generally, COX-1 is a constitutive or non-inducible form expressed in many tissues and important in tissue homeostasis. In the contrary, the inducible COX-2 is rapidly induced in inflammatory cells upon exposure to pro-inflammatory cytokine, tumor promoters, oncogenes and growth factors [3,4]. Lastly, COX-3, first identified by Chandrasekharan *et al.*, was shown to exhibit the catalytic activity and structure of COX-1 and COX-2, but to retain the intron 1 that is not exist in COX-1. However, the precise roles and functions of COX-3 are yet to be elucidated [5].

The prolonged synthesis of PGE_2_ or overexpression of COX-2 had been linked with numerous inflammatory disorders, such as asthma, oseteoarthritis, rheumatoid arthritis, Alzheimer’s disease and so on, hence, a number of strategies have been designed to block the COX enzyme activity or its expression, ultimately inhibiting PGE_2_ synthesis to enhance disease management [6]. Of these, non-steroidal anti-inflammatory drugs (NSAID) are the most widely used pain killer in relieving the chronic pain associated with various inflammatory diseases. Due to the crucial role of constrictive COX-1 in gastro-protection, the non-selective inhibitory action of NSAID on both COX-1 and COX-2 enzyme results in high degree of gastrointestinal toxicity and ulceration. This phenomenon prompted an investigation aimed at discovering alternative and selective drug targeting on COX-2 with minimal gastrointestinal-related side effects [7,8].

A number of studies have shown the great chemical and pharmacological potential of synthetic curcumin analogues that demonstrate antioxidant, anti-proliferation, anti-angiogenesis, anti-tumour, and anti-inflammatory properties [9,10,11,12]. We previously also reported that the curcumin derivative BDMC33 [2,6-bis(2,5-dimethoxybenzylidene) cyclohexanone] exhibited improved anti-inflammatory activities by inhibiting NO production in IFN-γ/LPS challenged macrophage cells [13]. In this study, we further demonstrate the immunomodulatory action of BDMC33 on inhibition of PGE_2_ synthesis via suppression of COX-2 in IFN-γ/LPS-challenged macrophage cells.

## 2. Results and Discussion

### 2.1. Effects of BDMC33 on PGE_2_ Secretion and Cell Viability

**Figure 1:**
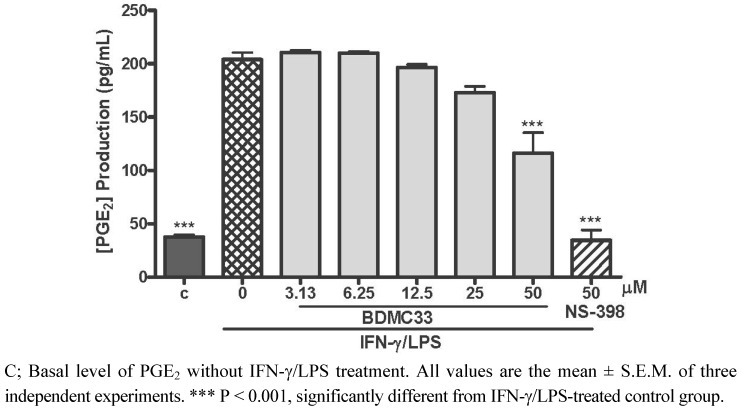
Effect of BDMC 33 on PGE_2 _production in IFN-γ/LPS-induced RAW 264.7 macrophages. Cells were stimulated with combination of IFN-γ/LPS and treated with increasing concentration of BDMC 33 for 18 to 20 h. PGE_2 _level was determined by using Cayman EIA kit. The IC_50_ was calculated at 47.33 ± 1.00 μM. NS-398 (50 µM) was used as positive control for PGE_2_ inhibition.

Regarding the enormous and widespread induction of COX-2 with consecutive formation of high amount of PGE_2_ in activated macrophage cells, this inflammatory mediator has been made responsible for various inflammatory disorders [14]. Therefore, inhibition of PGE_2_ synthesis by targeting the COX enzyme activities has served an important anti-inflammatory pharmacotherapy intervention over the past decades. In this study, the effect of BDMC33 on PGE_2_ synthesis in macrophage cells was determined. As demonstrated in Figure 1, a trace amount of PGE_2_ was detectable in the culture media at the mean concentration of 37.63 ± 1.97 pg/mL. The induction of RAW 264.7 cells into an inflammatory state by combination treatment of IFN-γ/LPS resulting in synthesis and secretion of PGE_2_ into culture media (203.94 ± 6.66 pg/mL). BDMC 33 showed a dose-related inhibition of PGE_2_ production, albeit with a low degree of inhibition. Significant inhibition of PGE_2_ production by BDMC 33 was only evident at 50 μM (P < 0.001) and the IC_50_ was calculated at 47.33 ± 1.00 μM. NS-398, a standard selective COX-2 inhibitor, was used as positive drug control and strongly inhibited PGE_2_ production (87.09 ± 8.26%) at 50 μM.

**Figure 2:**
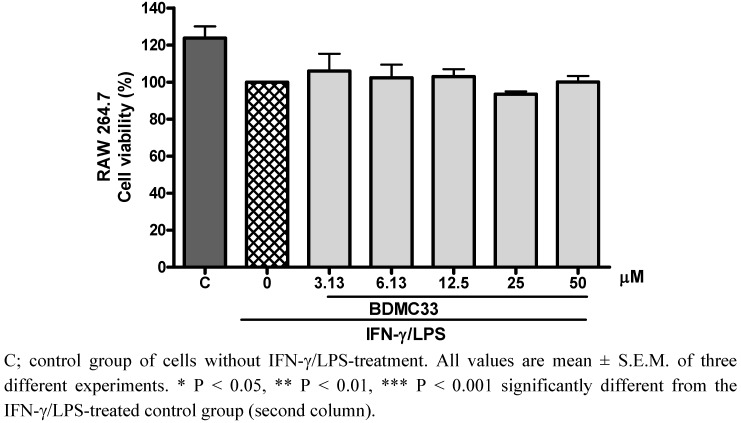
Effects of BDMC33 on RAW 264.7 cell viability. Cells were stimulated with combination of IFN-γ/LPS and treated with increasing concentrations of BDMC 33 for 18 to 20 h. Cell viability was determined by using a colorimetric MTT assay.

However, there was some concern as to whether the inhibitory action of BDMC 33 on PGE_2_ inhibitory activity was a false positive result from the cytotoxic effects of BDMC33, thus the cytotoxicity effect of BDMC33 was then evaluated by using the MTT assay. As shown in Figure 2, no significant alteration of the cell viability was found after BDMC33 treatment, thus excluding the possibility that the inhibitory action was due to the cytotoxicity of BDMC33.

### 2.2. Effects of BDMC33 on COX-2 Enzymatic Activity

**Figure 3:**
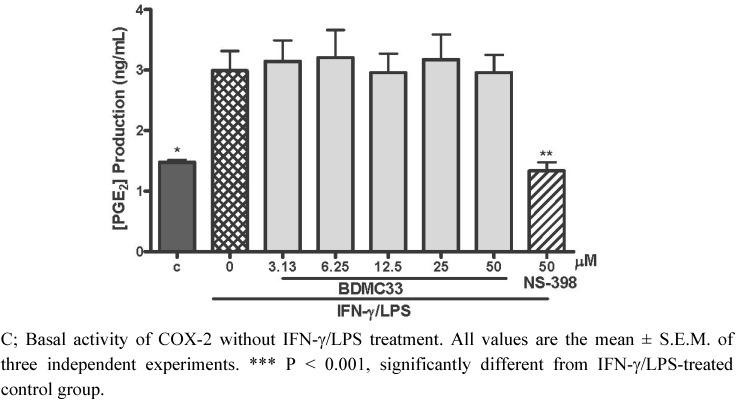
Effect of BDMC 33 on COX-2 activity in IFN-γ/LPS-induced RAW 264.7 macrophages. Cells were treated with IFN-γ/LPS for 6 h, followed by the treatment of increasing concentrations of BDMC 33 for another 30 min. Exogenous substrate of COX-2 enzyme (100 μM of arachidonic acid) was added and incubated for 15 min. NS-398 (50 µM) was used as positive control for COX-2 inhibition. PGE_2_ level in media was determined by the Cayman EIA kit.

In order to investigate the PGE_2 _inhibitory action of BDMC 33 via inhibition of COX-2 activity, the macrophages were induced for 6 h to up-regulate the COX-2 expression prior to the BDMC 33 treatment. The exogenous substrate (arachidonic acid) was added to determine the effect of BDMC 33 upon cellular COX-2 enzyme activity by evaluating the PGE_2 _level in media. As demonstrated in Figure 3, 1.48 ± 0.02 ng/mL of PGE_2_ is constitutively generated in the control group and IFN-γ/LPS treatment caused a significant induction of PGE_2_ generation (2.99 ± 0.55 ng/mL). The BDMC treatment did not show any significant alternation upon COX-2/arachidonic acid-generated PGE_2_ at any concentration tested. The PGE_2_ inhibitory action without alteration of COX-2/arachidonic acid-generated PGE_2 _secretion in macrophages thus suggests BDMC33 is not a potent COX-2 enzyme inhibitor. NS-398 as positive drug control shown significant inhibition on COX-2/arachidonic acid-generated PGE_2_ (58.78 ± 8.60%) at 50 μM.

### 2.3. Effects of BDMC33 on COX Expression

**Figure 4:**
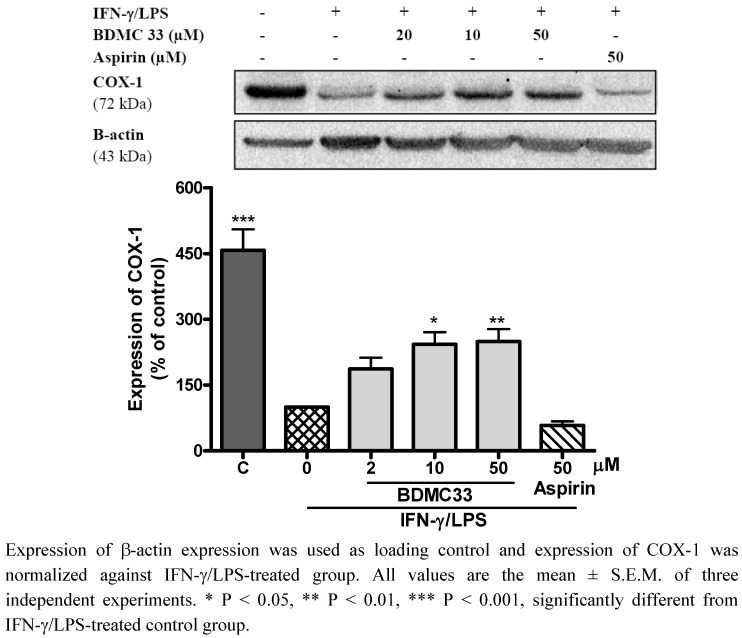
Effects of BDMC 33 on COX-1 expression of IFN-γ/LPS induced RAW 264.7 macrophages. Cells were stimulated for 20 h with combination of IFN-γ/LPS and treated with increasing concentration of BDMC 33. Whole cells lysate were assayed for COX-1 expression by using Western blotting. Aspirin served as positive drug control for COX-1 expression.

As BDMC33 did not show any significant inhibition on COX enzymatic activity, the PGE_2_ inhibitory activity of BDMC33 was investigated at the protein expression level. Hence, the effects of BDMC 33 on the of the isoforms of cyclooxygenase (COX) were examined. As shown in Figure 4, COX-1 protein was constitutively expressed in non-stimulated macrophages, but the expression level was downregulated after the IFN-γ/LPS treatments. According to Matsurra *et al.*, exposure of IFN-γ to normal human epidermal keratinocyte cells causes the gradual reduction of COX-1 expression [15]. Similarly, LPS treatment also found to inhibit constitutive COX-1 expression in pulmonary artery endothelium cells and rat tissue [16,17]. In agreement with previous reports, we suggest that macrophages stimulated by IFN-γ or/and LPS enable the attenuation of the constitutive COX-1 expression. Interestingly, BDMC 33 attenuated the downregulatory action of IFN-γ/LPS through sustaining the COX-1 expression in dose dependent manner. Aspirin a non-selective COX inhibitor used as a positive drug control, showed suppression of COX-1 expression in activated macrophages (40.90 ± 6.27%). As compared with NSAIDs (such as aspirin), the use of BDMC33 as an anti-inflammatory agent might overcome or reduce the gastro-related side effects through immunomodulatory action on COX-1 expression, which is crucial for gastroprotection.

**Figure 5:**
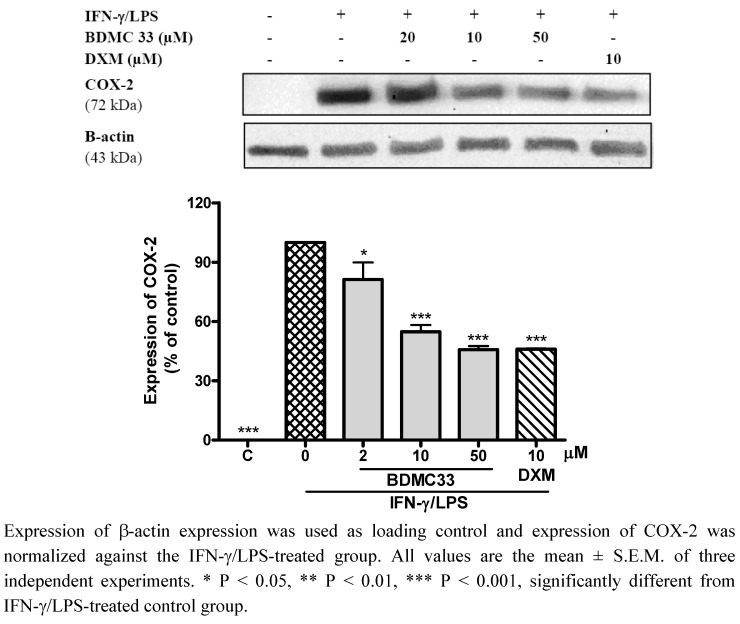
Effects of BDMC 33 on COX-3 expression of IFN-γ/LPS induced RAW 264.7 macrophages. Cells were stimulated for 20 h with combination of IFN-γ/LPS and treated with increasing concentration of BDMC 33. Whole cells lysate were assayed for COX-2 expression by using Western blotting. Dexamethasone (DXM) served as positive drug control for COX-2 expression.

It is well-known that challenges to macrophages with various stimuli result in elevated PGE_2 _secretion through induction of COX-2 expression [18]. In accordance with our results (Figure 5), the inducible COX-2 protein was absolutely undetectable in non-stimulated macrophages, but COX-2 was highly expressed and following induction of IFN-γ/LPS, BDMC 33 displayed a dose-dependent downregulatory action on COX-2 expression. Dexamethasone, a steroidal inflammatory agent, showed significant suppression of COX-2 expression (42.09 ± 5.92%) in IFN-γ/LPS-treated macrophages. A study conducted by Simmons *et al.* revealed that the expression of COX is chiefly regulated at the transcriptional level. The constitutive properties of COX-1 is contributed by the Sp-1 cis-regulatory element and lacking of TATA box in the COX-1 promoter, whereas the promoter of COX-2 contains a canonical TATA box and several inducible enhancer elements, such as cAMP response element (CRE), CCAAT enhancer-binding protein (C/EBP) and NF-κB [19]. Interestingly, Ye and Liu have reported the *in vivo* effects of LPS to up-regulate the inducible transcriptional factor activities of NF-κB and CREB, but down-regulate constitutively the transcriptional factor activities of SP-1 and AP-2 [20]. Based on these observations, we believe that the immunomodulatory action of BDMC33 on differential COX expression in IFN-γ/LPS-treated macrophage is mainly attributable to interference with these transcriptional factor activities and upstream signaling molecules.

## 3. Experimental

### 3.1. Materials

The following reagents were obtained commercially: Antibiotics (5,000 U/mL penicillin and 5,000 μg/mL streptomycin), Dulbecco’s Modified Eagle’s Medium (DMEM) from Flowlab^TM^ (North Ride, Australia); Foetal bovine serum from iDNA Biotechnology Bte Ltd (Singapore); recombinant mouse IFN-γ from eBioscience Inc. (San Diego, CA, USA); *Escherichia coli* (strain 055:B5), 3-(4,5-dimethylthiazol-2-yl)-2,5-diphenyl tetratzolium bromide (MTT) from Fluka Chemie GmbH (Buchs, Switzerland); dimethyl sulfoxide (DMSO) from Sigma Chemical Co. (St. Louis, MO, USA); Rabbit polyclonal against mouse COX-1 and COX-2 antibodies, PGE_2 _EIA kit from Cayman Chemicals (Ann Arbor, M, USA); HRP conjugated anti-β-actin from Santa Cruz Biotechnology (Santa Cruz, CA, USA); polyvinylidene fluoride (PVDF) membrane, HRP conjugated donkey anti-rabbit IgG and Enhanced Chemiluminescence Western Blotting Reagent (ECL) were purchased from Amersham Bioscience UK Ltd. (Buckinghamshire, UK).

### 3.2. Synthesis of 2,6-Bis(2,5-dimethoxybenzylidene)cyclohexanone (BDMC33)

**Scheme 1:**
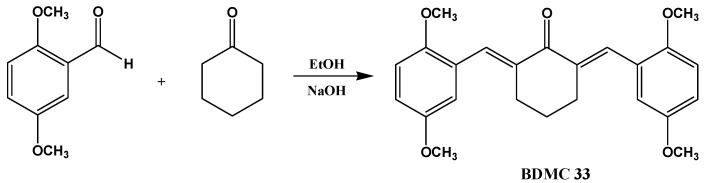
Chemical structure and synthesis of BDMC33.

BDMC33 or 2,6-bis(2,5-dimethoxybenzylidene)cyclohexanone was chemically synthesized in the Institute of Bioscience, Universiti Putra Malaysia (UPM) as described previously (Scheme 1) [13].

### 3.3. Cell Culture

The murine macrophages-like cell line (RAW 264.7) from the European Collection of Cell Cultures (Porton Down, UK) were maintained in DMEM supplemented with 10% FBS, 4.5 g/L glucose, sodium pyruvate (1 mM), L-glutamine (2 mM), streptomycin (50 μg/mL) and penicillin (50 U/mL) at 37 °C and 5% CO_2_. When RAW 264.7 cells reached confluency of 80–90%, the cells were scraped out and centrifuged at 110 × *g* in 4 °C for 10 min. The cell viability of cultured cells used in the assay was always >95% as determined by trypan blue dye exclusion.

### 3.4. Cell Stimulation and Treatment

RAW 264.7 (4 × 10^5^ cells/well) were seeded into a tissue culture grade 96-well plate except for blank and incubated for 2 h at 37 °C, 5% CO_2_ for cell attachment. The attached cells were activated with 100 U/mL of recombinant IFN-γ and 5 μg/mL of LPS with or without presence of BDMC33 at a final volume of 100 μL/well. DMSO was used as vehicle to add BDMC33 into the culture medium and the final concentration of DMSO was 0.1% in all cultures. Cells were then incubated at 37 °C, 5% CO_2_ for 17–20 h. The level of PGE_2 _was determined by using PGE_2_ EIA kit.

### 3.5. Cell Viability

The cytotoxicity of BDMC33 on cultured cells was determined by assaying the reduction of MTT reagents to formazan salts. After treatment, the supernatant of 96-wells plate containing cells were removed and MTT reagents (0.05 mg/mL) were added into each well. The cells were incubated in 37 °C for 4 h and the formazan salts were dissolved by adding 100% DMSO. The absorbance was then measured at 570 nm on a SpectraMax Plus Microplate reader (Molecular Devices Inc., Sunnyvale, CA, USA).

### 3.6. Determination of PGE_2_

The cell culture supernatants were collected and analyzed for PGE_2_ secretion PGE_2_ EIA kits (Cayman Chemical, Ann Arbor, MI, USA). The protocols provided by the manufacturers were followed to the detail. The data was acquired using a SpextraMax Plus microplate reader (Molecular Device, Sunnyvale, CA, USA). The concentration of PGE_2_ for each sample was calculated from their respective standard curves.

### 3.7. Indirect Determination of Endogenous COX-2 Activity

RAW 264.7 cells (4 × 10^5^ cells/well) were seeded into a tissue culture grade 96-well and triggered with IFN-γ/LPS for 6 h. Then, the spend media were removed and washed three times with 200 µL of media without IFN-γ/LPS. The 50 µL of serial concentration diluted BDMC 33 was added into the plate and incubated for a further 30 min in 37 °C. The exogenous substrate of arachidonic acid (50 µL) added for 15 min to produce sufficient substrate for measuring COX-2 enzyme activities. Lastly, the PGE_2_ concentration of supernatants was determined by PGE_2_ EIA kit.

### 3.8. Western Blotting Analysis of COX Expression

RAW 264.7 cells were induced with IFN-γ/LPS as described earlier and treated with different concentrations of BDMC33. To prepare the whole cell extract of RAW 264.7, the cells were scraped out from culture flask and washed three times with ice-cold Tris-sucrose washing buffer. Then, the cell pellets were resuspended in lysis buffer (0.5% Triton-X, 2 mM EDTA, 2 mM PMSF, 2 ng/μL pepstatin A, 1 µg/mL leupeptin, 1 µg/mL apotinin, 10 mM Na_3_VO_4_, in Tris-HCl, pH 7.5) and incubated in ice for 30 min. Following incubation, the cells were sonicated at 20 Hz for 30 s. Then the cells were incubated for 20 min on ice and centrifuged at 25, 150 × *g* at 4 °C for 30 min. The supernatants collected and protein content was measured using Bradford assay. In order to determine the expression of COX, equal amounts of protein (20 μg) were electrophoresed in a 10% SDS-PAGE and blotted onto a PVDF membrane (Amersham). Membrane were blocked for 1 h at room temperature in blocking buffer (0.3% BSA in PBST) and then incubated with primary antibodies for 1 h. The primary antibodies were used: rabbit polyclonal antibodies against COX-1 (1:5,000), COX-2 (1:5,000) and HRP conjugated β-actin (1:2,500). After several washes with PBST, HRP-conjugated donkey anti-rabbit IgG (1:1,000–1:2,000) were added for further 1 h incubation. The proteins were detected by using ECL blotting reagent according to the manufacturer’s instruction. The image captured using VersaDoc Imaging Device (BioRad, Hercules, CA, USA) and relative volumes of bands were measured by Quantity One software.

### 3.9. Statistical Analysis

Statistical analysis were performed using one–way analysis of variance (ANOVA) followed by Dunnett test *post hoc* for multi-group comparison test using GraphPad Prism (GraphPad Prism Software Inc., La Jolla, CA, USA). Statistical significance of differences between groups was accepted at *P* < 0.05.

## 4. Conclusions

In conclusion, we have demonstrated the immunomodulatory action of BDMC33 on PGE_2 _inhibition and differential expression of COX isoforms in IFN-γ/LPS-treated macrophage cells. The immunomodulatory effect of BDMC33 denotes a possible use as a future anti-inflammatory agent with minimal gastro-related side effects. However, further studies are needed to identify the molecular target(s) and signaling pathway that contribute the anti-inflammatory activities of BDMC33 on differential expression of COX isoform in macrophage cells. 
